# Extremophiles Breakthrough: Hot Topics and Current Issues in Their Isolation, Identification, and Biotechnological Applications

**DOI:** 10.3390/life15030434

**Published:** 2025-03-10

**Authors:** Martina Aulitto, Giovanni Gallo

**Affiliations:** 1 Department of Biology, University of Napoli Federico II, Complesso Universitario Monte Sant’Angelo, 80126 Napoli, Italy; 2 Division of Microbiology, Faculty of Biology, Ludwig-Maximilians-Universität München, 82152 Martinsried, Germany; giovanni.gallo@lmu.de

Extremophiles—organisms adapted to survive and thrive under extreme conditions—have fascinated scientists for decades, providing valuable insights into life’s resilience and offering untapped potential for technological innovation [1,2]. These extraordinary organisms inhabit some of the harshest environments on Earth, from Antarctic ice to deep-sea hydrothermal vents and hypersaline lakes. Their ability to overcome environmental extremes has made them models for studying evolution, adaptation, and the biochemical basis of life. Furthermore, extremophiles are emerging as a cornerstone of biotechnological advancements, with applications in medicine, industry, environmental management, and even space exploration [3,4,5]. This Special Issue highlights pivotal studies that demonstrate the diverse roles that extremophiles play in advancing scientific knowledge and addressing real-world challenges. From exploring their resilience to uncovering their industrial and astrobiological potential, these studies illustrate how extremophiles are shaping our understanding of biology and our approach to solving global problems.

The first contribution in this Special Issue sheds light on heat-resistant *Escherichia coli* (XHR *E. coli*), which harbors a genetic element known as the transmissible locus of stress tolerance (tLST). These bacteria can withstand heat treatments typically used during meat processing and cooking, posing a risk of contamination even after standard antimicrobial interventions. To address this issue, researchers developed optimized protocols for screening tLST genes by using multiplex PCR and isolating XHR *E. coli* from meat sources [6]. These methods consider the influence of heat exposure modes on the reliability of detection, achieving greater concordance between the genetic and phenotypic characteristics of XHR *E. coli.* This study emphasizes the need for tailored microbial control strategies in the meat industry, as conventional heat treatments may no longer suffice against these resilient strains. Beyond food safety, this work offers broader implications for understanding microbial survival strategies. The genetic mechanisms that enable heat resistance in XHR *E. coli* exemplify how microorganisms adapt to environmental stressors, providing insights into extremophile biology that could inform future research and applications.

Antarctica, one of the most isolated and extreme environments on Earth, represents a unique frontier for microbial discovery. Therefore, one contribution in this Special Issue explores the microbial composition of various environments near the Bulgarian Antarctic Base, “St. Kliment Ohridski”, on Livingston Island. Employing amplicon-based metagenomics targeting 16S rRNA and ITS2 regions, researchers uncovered a diverse array of bacterial, fungal, and archaeal communities [7]. The findings reveal a preponderance of taxa such as *Oxyphotobacteria*, *Bacteroidia,* and *Gammaproteobacteria*, alongside an intriguing dominance of an uncultured cyanobacterial taxon within the family *Leptolyngbyaceae*. Environmental factors, such as penguin activity, were shown to shape microbial compositions, particularly in a lagoon near the research base. Despite the abundance of bacterial taxa, fungal reads were predominantly unclassified, highlighting the vast unknown biodiversity in these regions. Meanwhile, archaeal abundance was minimal, dominated by groups such as *Woesearchaeales* and *Candidatus Nitrosopumilus*. This study not only contributes to cataloging Antarctic microbial diversity, but also highlights the biotechnological potential of these organisms. Cold-adapted enzymes and other biomolecules from Antarctic microbes could have applications in medicine, food preservation, and industrial processes.

Extremophiles are increasingly recognized for their role in producing robust enzymes capable of operating under challenging industrial conditions. One manuscript in this Special Issue introduces a novel type II L-asparaginase from a halotolerant strain of *Bacillus subtilis* CH11, isolated from the Chilca salterns in Peru [8]. L-asparaginases are widely used in cancer therapy and the food industry, making the discovery of more stable and efficient variants highly desirable. The enzyme, expressed in a heterologous *Escherichia coli* system, exhibited remarkable thermal stability, with optimal activity at pH 9.0 and 60 °C, and a half-life of nearly four hours at this temperature. Its activity was significantly enhanced by ions such as potassium and calcium, as well as reducing agents, suggesting its potential for diverse industrial applications. Furthermore, its kinetic properties demonstrate a balance of efficiency and substrate affinity, reinforcing its suitability for biotechnological use. This study underscores the value of extremophiles as sources of industrially relevant enzymes. Halotolerant organisms offer a reservoir of biomolecules tailored to withstanding high salinity and temperature, conditions that mirror many industrial processes.

While extremophiles are often associated with surface extremes, subterranean environments also harbor unique microbial communities. Therefore, one contribution in this Special Issue explores microbial diversity within the cave, combining long-term sampling and microcosm experiments to examine community composition and colonization dynamics [9]. Researchers found distinct prokaryotic communities across different cave zones, with specialized taxa adapting to localized energy and nutrient conditions. Notably, within a year, various minerals incubated in cave waters were colonized by specific microbial communities, demonstrating rapid adaptation to environmental opportunities. These findings highlight the ability of extremophiles to thrive and diversify even in resource-limited settings. Movile Cave serves as a model system for studying microbial ecology in extreme environments, providing insights into life’s adaptability. It also raises intriguing questions about the potential for life in similarly isolated and chemically unique habitats beyond Earth. Moreover, another study in this Special Issue explores bacterial communities in the copper mine of Wettelrode, Germany, revealing that disused copper mines serve as fascinating sites for the development of extremophilic bacteria, particularly halophiles [10]. Samples from the copper seam and the mine tunnel floor contained substantial numbers of halophilic bacteria, with site-specific variations. The presence of many halophilic genera shows similarities to microbial communities found in drainage from industrial copper mine tailings and potash mine efflux, with some analogies of preindustrial saline ash deposits. The study highlights how the highly saline and sulfidic environment of the copper ore shapes microbial diversity, leading to a high prevalence of sulfur-related metabolisms. Notably, the surface of the copper seam was dominated by sulfur-oxidizing bacterial strains, emphasizing the influence of local geochemical conditions on microbial adaptation. These findings provide insights into microbial ecosystems in extreme environments with potential applications in bioremediation and biotechnology.

Extremophiles are not just subjects of scientific curiosity, but also profoundly affect our understanding of life’s origins and resilience. Another review in this Special Issue reflects on the evolutionary strategies employed by extremophiles to survive in harsh environments [11]. From genome modifications to protein redesigns and membrane adaptations, these organisms demonstrate a remarkable capacity for innovation. Such adaptations have implications for astrobiology, as extremophiles provide a framework for imagining how life might arise and persist under extraterrestrial conditions. Moreover, their resilience could inform strategies to address modern challenges, such as developing bio-based solutions for climate change mitigation and environmental restoration. 

Finally, another insightful review addresses the recent advancements in extremophile research, focusing on their applications in enzyme production, bioplastics, environmental management, and space exploration [12]. The unique biological mechanisms that enable extremophiles to endure extreme conditions also make them ideal candidates for solving industrial and environmental problems. For example, extremophile-derived enzymes can enhance biocatalysis under conditions where the conventional enzymes fail, while their metabolic pathways offer blueprints for sustainable materials and bioenergy production. In space exploration, extremophiles are central to developing life-support systems and understanding planetary habitability. Their ability to survive radiation, desiccation, and extreme temperatures positions them as models for life in extraterrestrial environments.

In conclusion, the studies presented in this Special Issue reflect the growing importance of extremophiles in both fundamental and applied sciences. Researchers are opening new frontiers in biotechnology, environmental science, and astrobiology by exploring their diversity, understanding their adaptations, and harnessing their biochemical tools. Looking ahead, interdisciplinary collaborations will be crucial for unlocking the full potential of extremophiles [13]. Advances in genomics, synthetic biology, and systems biology offer exciting opportunities to engineer extremophilic traits for tailored applications. Moreover, the continued exploration of extreme environments—on Earth and beyond—will undoubtedly reveal new extremophiles and novel adaptations, further expanding the horizons of science and technology. As we continue to push the boundaries of knowledge, extremophiles remind us of life’s remarkable capacity to adapt and thrive, even in the most inhospitable corners of the universe.
